# Identification of *Cucumber mosaic resistance 2* (*cmr2*) That Confers Resistance to a New *Cucumber mosaic virus* Isolate P1 (CMV-P1) in Pepper (*Capsicum* spp.)

**DOI:** 10.3389/fpls.2018.01106

**Published:** 2018-08-21

**Authors:** Seula Choi, Joung-Ho Lee, Won-Hee Kang, Joonyup Kim, Hoang N. Huy, Sung-Woo Park, Eun-Ho Son, Jin-Kyung Kwon, Byoung-Cheorl Kang

**Affiliations:** ^1^Department of Plant Science, Plant Genomics and Breeding Institute, Research Institute of Agriculture and Life Sciences, Seoul National University, Seoul, South Korea; ^2^RDA-Genebank Information Center, Jeonju, South Korea

**Keywords:** *Cucumber mosaic virus* (CMV) resistance, molecular mapping, bulked segregant analysis (BSA), amplified fragment-length polymorphism (AFLP), Affymetrix array, germplasm screening

## Abstract

*Cucumber mosaic virus* (CMV) is one of the most devastating phytopathogens of *Capsicum*. The single dominant resistance gene, *Cucumber mosaic resistant 1* (*Cmr1*), that confers resistance to the CMV isolate P0 has been overcome by a new isolate (CMV-P1) after being deployed in pepper (*Capsicum annuum*) breeding for over 20 years. A recently identified Indian *C. annuum* cultivar, “Lam32,” displays resistance to CMV-P1. In this study, we show that the resistance in “Lam32” is controlled by a single recessive gene, *CMV resistance gene 2* (*cmr2*). We found that *cmr2* conferred resistance to CMV strains including CMV-Korean, CMV-Fny, and CMV-P1, indicating that *cmr2* provides a broad-spectrum type of resistance. We utilized two molecular mapping approaches to determine the chromosomal location of *cmr2*. Bulked segregant analysis (BSA) using amplified fragment-length polymorphism (AFLP) (BSA-AFLP) revealed one marker, cmvAFLP, located 16 cM from *cmr2*. BSA using the Affymetrix pepper array (BSA-Affy) identified a single-nucleotide polymorphism (SNP) marker (Affy4) located 2.3 cM from *cmr2* on chromosome 8. We further screened a pepper germplasm collection of 4,197 accessions for additional CMV-P1 resistance sources and found that some accessions contained equivalent levels of resistance to that of “Lam32.” Inheritance and allelism tests demonstrated that all the resistance sources examined contained *cmr2*. Our result thus provide genetic and molecular evidence that *cmr2* is a single recessive gene that confers to pepper an unprecedented resistance to the dangerous new isolate CMV-P1 that had overcome *Cmr1*.

## Introduction

Plant viruses cause significant losses of crop yield and quality worldwide (Kang et al., 2005). Pepper (*Capsicum* spp.) production is hampered by numerous plant pathogens, including more than 60 viruses (Kenyon et al., 2014). Control of such viral pathogens can be challenging due to their broad host range and the large number of insect vectors. The use of resistant cultivars is the most effective, and in many cases the only, strategy to limit plant viral diseases (Gray and Moyer, 1993). Therefore, it is a priority to identify virus resistance genes and dissect their resistance mechanisms in order to introduce strong and durable resistance into cultivars.

Virus resistance in plants can be classified into several categories that include the RNA silencing response, pathogen-associated molecular pattern (PAMP)-triggered immunity (PTI), resistance (R) protein-mediated resistance, and recessive resistance caused by mutations of host factors (Nakahara and Masuta, 2014; Gouveia et al., 2017; Islam et al., 2017). In the RNA silencing response, the antiviral RNA surveillance system in the host is triggered by double-stranded viral RNA (Hammond et al., 2001; de Ronde et al., 2014; Islam et al., 2017). The elicited antiviral RNA silencing machinery (RNAi) defends against all RNA and DNA viruses (Incarbone and Dunoyer, 2013), although it is a relatively slow process and does not render complete viral resistance. In contrast, the PTI-mediated resistance is the first layer of innate immunity associated with the conserved structural motifs of pathogen, known as PAMPs (Calil and Fontes, 2016). Concerning the viral defense mechanism, a few studies have been reported for the PTI response. The signaling modules by the anti-viral PTI are shown to be a mimicry of those for PTI responses against non-viral pathogens. For instance, it has been reported that a transmembrane immune receptor can activate host translational suppression to defend the viral attack (Zorzatto et al., 2015). Recent evidence indicates that PTI responses to viruses in Arabidopsis are elicited by dsRNA produced during virus replication and that the dsRNA response pathway involves SOMATIC EMBRYOGENESIS RECEPTOR-LIKE KINASE 1 (SERK1) (Niehl et al., 2016). Previous studies indicated that PTI responses to plant viruses in Arabidopsis also depend on BRASSINOSTEROID INSENSITIVE1 (BRI1)-ASSOCIATED RECEPTOR KINASE1 (BAK1/SERK3) (Kørner et al., 2013).

In R protein-mediated resistance, single dominant R proteins specifically perceive intercellularly secreted effectors of pathogens and activate a robust defense response (effector-triggered immunity) (Win et al., 2012). The majority of plant R genes encode proteins with nucleotide-binding and leucine-rich-repeat domains (Jones and Dangl, 2006). The strategy of dominant R protein-mediated resistance is effective against viruses as well as other non-viral pathogens. More than 20 anti-viral R genes with dominant inheritance have been characterized (de Ronde et al., 2014; Islam et al., 2017). Recognition of effectors by R proteins is proposed to occur through direct ligand-receptor interactions (Flor, 1971) or indirect interactions (Jones and Dangl, 2006; van der Hoorn and Kamoun, 2008; Osswald et al., 2014).

Another class of resistance, recessive resistance, arises when host factors essential for virus infection are altered or absent. Notably, nearly half of the plant genes that confer resistance to plant viruses are inherited in a recessive manner (Jørgensen, 1992), and recessive resistance appears to be a more common defense mechanism for viruses than for other non-viral pathogens (Diaz-Pendon et al., 2004). Conceptually, recessive resistance seems to be more durable and less strain-specific than dominant forms of resistance (Kang et al., 2005). For instance, the eukaryotic translation initiation factor 4E (eIF4E) and 4G (eIF4G) in eukaryotes are essential host factors required for infection by many viruses including Potyvirus and mutations of these gene results in recessive resistance in crop plants (Gal-On et al., 2000; Kang et al., 2005; Truniger and Aranda, 2009; Bastet et al., 2017). In another example of recessive resistance, Pelo was confirmed as a *Tomato yellow leaf curl virus* (TYLCV) resistance-associated gene, *ty5* (Lapidot et al., 2015). In a resistant *ty5* tomato line, impaired functions of Pelo suppressed viral infection associated with the protein synthesis and ribosome recycling-phase.

*Cucumber mosaic virus* (CMV), a member of the genus *Cucumovirus* in the family *Bromoviridae*, is one of the most destructive viruses in temperate regions. CMV has an exceptionally wide host range, infecting more than 1,200 plant species, and is transmitted by more than 80 aphid species (Palukaitis et al., 1992; Palukaitis and García-Arenal, 2003). CMV infection is responsible for significant economic losses in many crops including peppers (Palukaitis et al., 1992). It has been demonstrated that a dominant gene of Arabidopsis, *RCY1*, confers resistance to the yellow strain of CMV (CMV-Y) in a gene-for-gene manner (Takahashi et al., 2002; Sekine et al., 2008). Upon CMV infection, a dominant R gene of common bean (*P. vulgaris* CMV RESISTANCE 1, *PvCMR1*), triggered systemic necrosis in *Nicotiana benthamiana* (Seo et al., 2006), demonstrating that the legume R gene can be functional across two plant families. In addition, mutation of Vacuolar Protein Sorting 41 protein was shown to confer resistance to CMV in melon (Giner et al., 2017). Apparently, development of CMV-resistant varieties has been an important goal for crop breeding.

Over the last few decades, various sources of resistance to CMV have been identified in *Capsicum*. Most sources display polygenic resistance controlled by multiple genes. These include *C. annuum* “Perennial” (Caranta and Palloix, 1996; Lapidot et al., 1997; Grube et al., 2000), *C. annuum* “Vania” (Caranta et al., 2002), *C. annuum* “Sapporo-oonaga” and “Nanbu-oonaga” (Suzuki et al., 2003), *C. frutescens* “BG2814-6” (Grube et al., 2000), *C. frutescens* “LS1839-2-4,” and *C. baccatum* “PI439381-1-3” (Suzuki et al., 2003; Kang et al., 2010). Diverse mechanisms that underlie the resistance in these varieties include the inhibition of viral replication, cell-to-cell movement, and long-distance movement of viral particles. Genetic analyses were carried out to identify the genomic regions controlling the resistance and revealed that the resistance was controlled by several QTLs in some varieties (Caranta and Palloix, 1996; Chaim et al., 2001; Caranta et al., 2002). Resistance associated with QTLs has a great advantage of combating multiple viral strains. However, it is much more difficult to introduce multiple resistance genes into a breeding system in comparison with single resistance genes.

*Cucumber mosaic virus* is one of the most recurrent viruses infecting pepper in South Korea (Choi et al., 2005). The Chinese *C. annuum* variety “Likeumjo” is resistant to CMV-P0 (Kang et al., 2010) and is a rare CMV resistance source controlled by a single dominant gene, *Cucumber mosaic resistant 1* (*Cmr1*). *Cmr1* is located on chromosome 2 and markers linked to *Cmr1* have been developed and utilized for breeding (Kang et al., 2010). After being deployed for more than 20 years, *Cmr1* was found to be overcome by a new CMV isolate, CMV-P1 (Lee et al., 2006).

In this study, we report a novel CMV resistance gene, *CMV resistance gene 2* (*cmr2*), which confers resistance to CMV-P1 in a recessive manner. To develop molecular markers and map *cmr2*, we performed bulked segregant analysis (BSA) with F_2_ plants by addressing amplified fragment-length polymorphisms (BSA-AFLP) and differential signal strength upon hybridization of DNA fragments to Affymetrix chips (BSA-Affymetrix) carrying pepper expressed sequence tag (EST) sequences (Hill et al., 2013). We could find *cmr2*-linked markers using these two methods. We also screened a collection of *Capsicum* germplasm for additional CMV-P1 resistance sources. The screened germplasm that showed resistance to CMV-P1 also contained *cmr2*, indicating that *cmr2* is a natural genetic source for resistance to CMV-P1 that is distinct from the dominant resistance gene *Cmr1* for the P0 isolate.

## Materials and Methods

### Plant Materials

An Indian *C. annuum* landrace “Lam32” (provided by Nongwoo Bio, Co., Ltd., South Korea) and the Korean cultivar “Jeju” were used as resistant and susceptible parents, respectively. A commercial *C. annuum* F_1_ hybrid “Bukang” containing *Cmr1* was used as a control plant. To study the inheritance pattern of the resistance gene in “Lam32,” F_1_ seeds were obtained by crossing “Lam32” to “Jeju.” An F_2_ population was generated by self-pollinating the F_1_ plants and was used for mapping the CMV resistance gene in “Lam32.”

To identify additional CMV-P1 resistance sources, a total of 4,197 pepper accessions kindly provided by the National Agricultural Plant Genebank of Rural Development Administration (Jeonju, South Korea) were used. Pepper accessions showing resistance to CMV-P1 were identified and crossed with “Jeju” to study the inheritance pattern of the resistance gene. For the allelism tests, all selected accessions exhibiting CMV-P1 resistance were crossed with “Lam32.” To harvest seeds, plants were grown in the greenhouse at Seoul National University Farm (Suwon, South Korea).

### Virus Inoculation

The virus inocula were kindly provided by Nongwoo Bio, Co., Ltd. (Yeoju, South Korea). Because the infectivity of CMV-P1 was better with *Nicotiana rustica* than pepper, the CMV-P1 strain was inoculated and maintained in *N. rustica*. To create virus-containing inocula, infected *N. rustica* leaves (1 g) were harvested and ground on ice in 10 mL of 0.1 M phosphate buffer (pH 7.0). For each screening assay, we propagated the frozen inoculum in *N. rustica* 10–14 days before use. To avoid virus evolution the number of passages was reduced to a minimum by inoculating *N. rustica* plants always with the same original inoculum. The inoculum preparation was carried out on ice. *C. annuum* plants at the two-leaf stage were mechanically inoculated with CMV-P1 inocula on the cotyledons after dusting with Carborumdum #400 (Hayashi Pure Chemical, Co., Ltd., Japan). The inoculated plants were then rinsed with tap water for 10 min after inoculation. After 1 week, the inoculation process was repeated on the first true leaves to prevent their escape from infection. Inoculated plants were kept in a growth chamber in a 16-h light/8-h dark cycle at 23°C. To test the mutational effect that might have otherwise existed and broken the infectivity of CMV-P1, we inoculated the CMV-P1 isolate with Bukang F_1_ hybrid which has CMV-P0 resistance gene, *Cmr1*, and also tested for its infectivity before use in this study. At least five plants of each accession were inoculated for screening CMV-P1 resistance.

As to the constructs, the *Agrobactericum* harboring three CMV_FNY_ RNA genome constructs (pSNU1::CMV-RNA1, pSNU1::CMV-RNA2 tagged with green fluorescent protein (GFP), and pSNU1::CMV-RNA3) (Seo et al., 2009; Kang W.H. et al., 2012) was prepared and used for the inoculation. Three CMV constructs were mixed in a 1:1:1 ratio at OD_600_ of 1.0. CMV constructs were diluted in 10 mM MES/10 mM MgCl_2_ buffer at room temperature for 1 h. Pepper plants were infiltrated with the mixture of the constructs using a 1-mL syringe.

### Detection of CMV Accumulation

Double antibody sandwich (DAS)-enzyme-linked immuno-sorbent assay (ELISA) was used for CMV detection, following the manufacturer’s protocol (Agdia, Elkhart, IN, United States). Two leaf disks of leaf samples were collected at 25 days post-inoculation (dpi) for ELISA. Samples were measured at an absorbance of 405 nm in a Zenith 200 ELISA reader (Anthos, Eugendorf, Austria) (Kang H.K. et al., 2012) with a limitation set at 4. For statistical analysis, Duncan’s multiple tests were employed to compare with positive and negative controls. Three plants were grown and leaf samples collected three separate times to give three independent experimental replicates.

### BSA-AFLP

We used BSA-AFLP to locate *cmr2* in the pepper genome. Total DNA was extracted from the young leaves of plants using CTAB method (Hwang et al., 2009). The DNA extracts of 12 resistant and 13 susceptible plants of the F2 population derived from the cross between “Jeju × Lam32” were pooled for analysis. The DNA of selected 12 resistant plants and 13 susceptible plants from the cross between “Jeju × Lam32” were separately pooled for BSA. The AFLP polymerase chain reaction (PCR), genomic DNA digestion, adapter ligation, re-amplification, and selective amplification were performed as previously described with minor modifications (Vos et al., 1995). In brief, the pooled DNA was digested with EcoRI and MseI followed by ligation of adaptors to the overhanging sticky ends produced by the restriction enzymes. This was subjected to selective amplification of fragments based on the combination of 512 primer sets that recognize the specific adapter sequence plus specific nucleotides further inside the original DNA fragment given by the primer sequence elongations “ANN” or “CNN,” whereby “N” stands for a specific nucleotide in the primer. The select polymorphic data were applied to individuals to evaluate the correlation between a marker genotype and phenotype. The experimental procedures and analyses were described by Jo et al. (2016).

### BSA Using Affymetrix Chips

Bulked segregant analysis single-feature polymorphisms (SFPs) were identified using an Affymetrix array as described previously (Sim et al., 2009). For this analysis, 24 resistant and 8 susceptible plants in the F_2_ population derived from “Jeju × Lam32” were selected based on their phenotypes and separately pooled for the analysis. The bulked DNA (30 μg/array) was randomly fragmented into 100–200 bp using DNase I. The fragmented DNA samples were labeled as previously described (Liu et al., 2014). Bulked DNA samples was hybridized on the custom Affymetrix pepper chip array (Affymetrix, Santa Clara, CA, United States) with four biological replications. Probe sets on pepper chip array were based on EST sequences constructed from UC Davis (Hill et al., 2013). Probe signals were processed and analyzed based on a non-uniform drop in signal intensity for individual probes within a probe set (Li and Durbin, 2009). The R package^1^ was used to identify SFPs showing a Dstat value of ≥3 or ≤-3 (Borevitz et al., 2003; Gore et al., 2007) as described previously (Liu et al., 2014). EST sequences corresponding to the selected SFPs were identified from the first version of the *C. annuum* genome database^2^.

### High-Resolution Melting Marker Polymorphism Survey

Single-nucleotide polymorphism markers were used for the polymorphism survey between the two parental lines, “Jeju” and “Lam32.” SNPs were surveyed by high-resolution melting (HRM) analysis using a Rotor-Gene 6000 thermocycler (Qiagen, Germany). PCR was carried out in 20-μL reaction volumes with 50 ng genomic DNA as template, 1× HiPi buffer (ELPIS-Biotech, South Korea), 0.2 mM dNTPs, 500 mM each forward and reverse primer (Bioneer, South Korea), 1.5 μM SYTO9 (Invitrogen, United States), and 0.6 units home-made *Taq* DNA polymerase. PCR conditions were: initial denaturation at 94°C for 2 min followed by 35 cycles of 94°C for 30 s, 55°C for 1 min, and 72°C for 1 min. PCR for HRM analysis was carried out in 20 μL reaction volumes containing 20 ng of genomic DNA, 6 pmol of each primer, and 2× HRM master mix (Roche, Basel, Switzerland). The conditions of HRM were 94°C for 4 min followed by 45 cycles of 94°C for 10 s, 56°C for 30 s, and 72°C for 30 s.

### Kompetitive Allele-Specific PCR Marker Analysis

A Kompetitive Allele-Specific PCR (KASP) marker converted from HRM marker (Affy4) was designed for mapping *cmr2* on the “Jeju × Lam32 F_2_” map and for the allelism test of new CMV-P1 sources of resistance. Thermocycling and endpoint genotyping for the KASP assays were performed in a Roche LC480 (Roche Applied Science, Indianapolis, IN, United States). To genotype the segregating plant populations, 1 μL plant DNA (100 ng) was mixed with 5 μL KASP reaction mix and 0.14 μL gene-specific KASP primer mix in a total reaction volume of 10 μL. The entire setup was run in a 96-well qPCR plate and the thermal cycling conditions were as follows: hot start at 94°C for 15 min followed by 9 cycles of 94°C for 20 s and 61°C for 60 s. The annealing temperature was dropped at the rate of 0.6°C/cycle followed by 27 cycles at 94°C for 20 s and 55°C for 60 s, and 3 cycles of 94°C for 20 s and 57°C for 60 s. Post-melt cycles were at 57°C for 10 s and cooling to 20°C during plate reading. For signals with weak separation, five additional cycles of 94°C for 20 s and 57°C for 60 s were performed.

### Linkage Analysis of SNP-Based Markers

Single-nucleotide polymorphism markers showing polymor-phisms between the two parental lines were used for linkage analysis (**Table 1**). Linkage analysis of makers was performed using Carthagene software (de Givry et al., 2005). The Kosambi function was used to convert recombination values to genetic distance; a minimum LOD score of 3.0 and a maximum distance of 30 cM were used as the threshold value.

**Table 1:** Molecular markers linked to the *cmr2* gene.

Marker	Type	Primer sequence (5′–3′)	Product size (bp)	Mapping population	Recombinants/total number of F_2_ individuals
Affy4	KASP	FAM) CCGACTTCGAGCAAGCCTACAT	119	J × L F_2:3_	9/195
		HEX) CGACTTCGAGCAAGCCTACAG			
		Common) CGTCCTGACCCGCCTGCCAT			
IBP160	HRM	F) CTTGACGTTGGACCCATCAA	252	J × L F_2:3_	52/195
		R) TGGACGTTCCCAATCAGAGA			
cmvAFLP	HRM	F) TGCAGTTGGAGCAGAAGATG	242	J × L F_2:3_	64/195
		R) CATGGAAAGACTCCCAAGGAAC			

*“J × L F_*2:3*_,” *C. annuum* “Jeju” ×*C. annuum* “Lam32” 195 F_*2:3*_ population*.

## Results

### Broad-Spectrum CMV Resistance in “Lam32”

**FIGURE 1:**
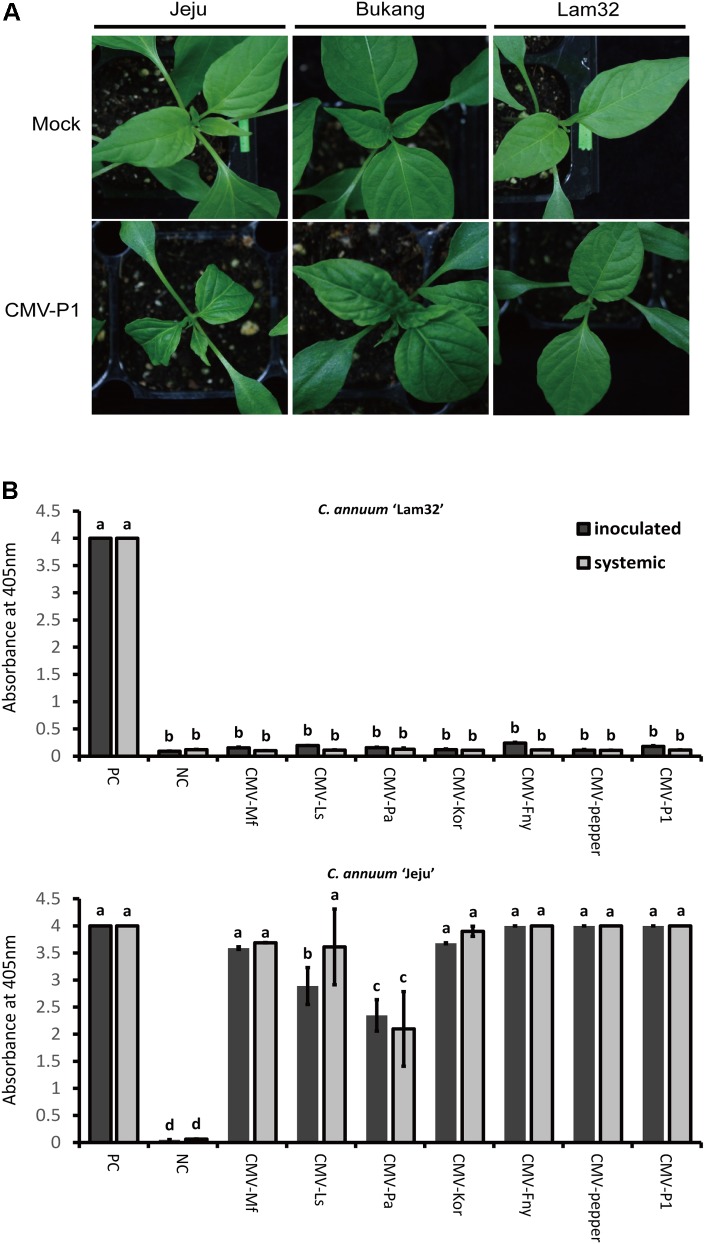
Disease response in pepper inoculated with seven CMV isolates. **(A)** CMV-P1 symptom of systemic leaves in resistant plant “Lam32” and susceptible plants “Jeju” and “Bukang” at 20 dpi. **(B)** Accumulation of CMV coat protein in inoculated leaves (cotyledon) and systemic leaves of peppers detected by ELISA assay (Absorbance at 405 nm). Positive control (PC) tubes in Agdia ELISA kit and Negative control (NC) as mock-inoculated leaf samples were used to compare with seven CMV isolates (Mf: CMV-MSf; Ls: CMV-Ls; Pa: Kor: CMV-Kor; Fny: CMV-Fny; Pepper: CMV-pepper; P1: CMV-P1). Values of *y*-axes are the mean of triplicate for each biological sample pooled from three plants. Error bars represent the standard deviation of the mean absorbance value at 405 nm and different letters represent significant differences [analysis of variance (ANOVA); *P* ≥ 0.05].

Cotyledons of three *C. annuum* varieties “Jeju” (susceptible), “Bukang” (resistant to CMV-P0, used as a control), and “Lam32” (resistant to CMV-P1) were inoculated with CMV-P1. At 20 days post-inoculation (dpi), typical CMV responses, such as yellowing, wilting, and mosaic symptoms, were clearly visible on the systemic leaves of “Jeju” and “Bukang” (**Figure 1A**). The susceptibility of “Bukang” to CMV-P1 indicated that its resistance against CMV-P0 strains (CMV-Kor and CMV-Fny) mediated by *Cmr1* (Kang H.K. et al., 2012) was overcome by the P1 isolate (**Figures 1A,B**). By contrast, “Lam32” showed no discernable CMV symptoms (**Figure 1A**) (Kang W.H. et al., 2012). To substantiate this observation, we performed ELISA to examine viral accumulation with seven different CMV-P0 and P1 isolates. “Lam32” exhibited no virus accumulation for all CMV isolates tested in both inoculated and systemic leaves (**Figure 1B**). These results demonstrated that “Lam32” has resistance against CMV-P0 and CMV-P1.

To gain insight into the mechanisms underlying the CMV resistance conferred by *cmr2* in detail, CMV_FNY_ tagged with GFP in the modified binary vector pSNU1 (Seo et al., 2009) was used for inoculation of “Lam32” and “Jeju.” In “Jeju,” the GFP signal was observed in both epidermal and mesophyll cells at 6 dpi (**Supplementary Figures S1E,F**), whereas the GFP signal was not detected in “Lam32” except for the inoculated site (**Supplementary Figures S1G,H**). These results support the idea that the resistance of “Lam32” to CMV-P1 may be acquired, in part, by impairing the movement of the virus. Nevertheless, substantial investigations on more time-point experiments for cellular location of CMV-P1 would provide better insights into *cmr2* resistance mechanism in pepper.

### Inheritance of CMV Resistance in “Lam32”

To examine the inheritance pattern of CMV resistance in “Lam32,” we observed the segregation ratio for resistance in F_2_ and BC_1_ populations derived from a cross between “Lam32” and “Jeju.” Among the 129 F_2_ plants, the pattern of resistance segregated in a 1R (resistant): 3S (susceptible) ratio. The segregation ratio of a backcross population crossed with the susceptible parent (BC_S_, an F_1_ plant from the cross between “Lam32” and “Jeju” crossed with “Jeju”) was 0R:1S, whereas the segregation ratio in a backcross population crossed with the resistant parent (BC_R_, an F_1_ plant from the cross between “Lam32” and “Jeju” crossed with “Lam32”) was 1R:1S (**Table 2**). Our inheritance study thus demonstrated that the CMV-P1 resistance in “Lam32” is controlled by a single recessive gene, and we named this gene *cmr2*.

**Table 2:** Segregation of CMV resistance in the progeny of crosses between “Jeju” and “Lam32”.

Populations	Expected ratio (R:S)^a^	Observed frequency		*X*^2b^	*P*^c^
		R	S		
Jeju	0:1	0	15	–	–
LAM32	1:0	15	0	–	–
F_1_ “Jeju × Lam32”	0:1	0	20	–	–
F_2_ “Jeju × Lam32”	1:3	37	92	0.038	0.308
BC_1_F_1_ “(Jeju × Lam32) × Jeju”	0:1	0	50	–	–
BC_1_F_1_ “(Jeju × Lam32) × Lam32”	1:1	198	162	3.6	0.058

^a^*R, resistant plants; S, susceptible plants. ^*b*^Chi-square test. ^*c*^Probability value*.

### Chromosomal Location of *cmr2*

To determine the chromosomal location of *cmr2*, we conducted BSA-AFLP using 512 primer combinations. AFLP fragments were selected to examine the correlation between the resistance phenotype and the genotypes of the F_2_ individuals. Of the 512 tested markers, one AFLP fragment (cmvAFLP) showed specific polymorphisms that correlated with the expected genotype of the 12 resistant and 13 susceptible individuals (**Supplementary Figure S2A**). We cloned and sequenced the fragment and identified one SNP in the sequence, which was used to design an HRM marker for cmvAFLP that amplified a product of 242 bp (**Supplementary Figure S2B**). This HRM marker could discriminate between the three genotypes (homozygous resistant, heterozygous susceptible, and homozygous susceptible) based on the different melting curves (**Supplementary Figure S2C**).

We used the developed HRM marker for linkage analysis and mapped *cmr2* to chromosome 1 (or 8) of the “AC99” map (Livingstone et al., 1999). The precise localization of some markers on chromosome 1 (or 8) can be difficult due to a translocation event between chromosome 8 and 1 during the domestication of *C. annuum* (Wu et al., 2009). In line with this, nine markers linked to cmvAFLP were located on chromosome 1 and five markers on chromosome 8 (data not shown).

### Fine Mapping of CMV-P1 Resistance-Linked SNP Markers

Because our HRM marker was not completely linked to *cmr2*, we developed additional markers closely linked to *cmr2*. We conducted a BSA-SFP analysis using microarray technology. For this purpose, we selected 24 plants that were resistant and eight plants that were susceptible to CMV-P1 from “Jeju × Lam32” F_2_ population. A total of 881 contigs with significant signal differences between the resistant and susceptible trait were identified. We chose 68 contigs each representing one unique EST to avoid duplicates and utilized them to design HRM markers based on their SNPs (Affy1 to Affy68). Of the 68 markers, 29 markers exhibited polymorphism between “Jeju” and “Lam32” and were used to genotype 195 F_2_ plants generated from the “Jeju × Lam32” cross. The Affy4 marker derived from “CAPS_CONTIG.1005” was confirmed to be closely linked to *cmr2*. When this marker was converted to a KASP marker, it clearly showed polymorphisms that segregated with resistance and susceptiblity. In addition, sequence analysis of “CAPS_CONTIG.1005” revealed that *cmr2* is closely located in an rRNA-rich region.

**FIGURE 2:**
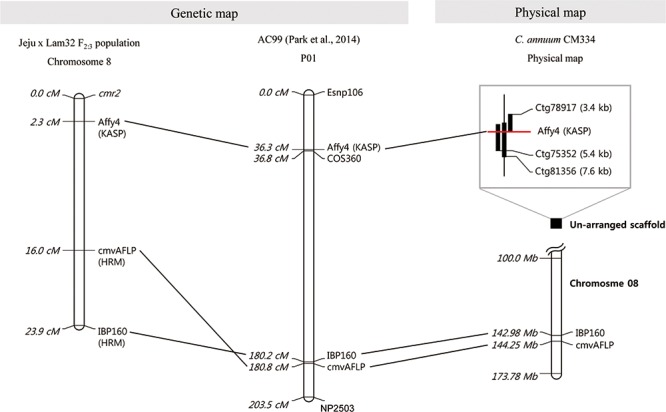
Genetic linkage map and physical map of *cmr2* linked markers. *Dotted lines* indicate commonly anchored markers among the maps. From the *left*, Genetic map “Jeju × Lam32 F_2:3_”, AC99 map (Park et al., 2014) and physical map *C. annuum* “CM334” (information of the Affy4 marker-linked scaffolds is presented in the *box*) (Hulse-Kemp et al., 2018).

Fine mapping was carried out with additional markers acquired from the “AC99” map (Park et al., 2014). We tested several markers closely linked to cmvAFLP to identify polymorphisms between “Jeju” and “Lam32,” and selected IBP160 as an additional marker. From the genetic and physical map constructed using three markers (cmvAFLP, IBP160, and Affy4), cmvAFLP and IBP160 were aligned on chromosome 8 of *C. annuum* CM334 (Hulse-Kemp et al., 2018), and Affy4 was located at 2.3 cM from *cmr2* (**Figure 2**).

### Identification of New CMV-P1 Resistance Sources

**FIGURE 3:**
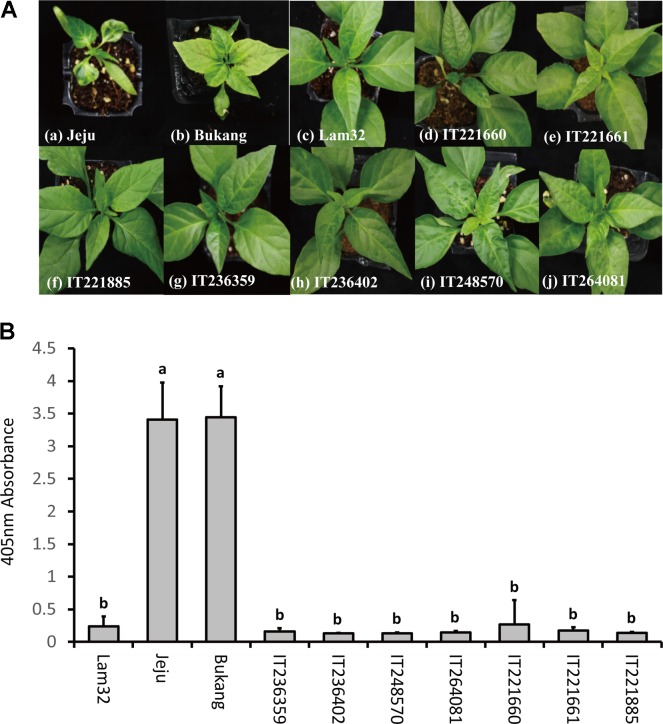
Disease responses to CMV-P1 infection at 25 dpi. **(A)** “Jeju” **(a)** and “Bukang” **(b)** are susceptible controls, “Lam32” **(c)** is a resistant control. Accessions **(d–j)** had no CMV symptoms (mosaic or cholorotic symptoms) in systemic leaves. **(B)** Accumulation of CMV coat protein in systemic leaves of CMV-P1 resistant accessions. Values of *y*-axes are the mean of triplicate for each biological sample pooled from three plants. Error bars represent the standard deviation of the mean absorbance value at 405 nm and *different letters* represent significant differences (ANOVA; *P* ≥ 0.05).

A total of 4,197 accessions of *Capsicum* germplasm were screened for CMV-P1 resistance with susceptible controls “Jeju” and “Bukang,” and the resistant control “Lam32.” The cotyledons from 8- to 10-day-old seedlings were inoculated with CMV-P1 and symptoms were observed every other day. From the initial screening of the *Capsicum* germplasm, we selected 21 candidate accessions that appeared resistant. These included 18 accessions of *C. annuum* and three accessions of *C. frutescens* (**Supplementary Table S1**). Both inoculated and systemic leaves of the 21 resistant candidate accessions were harvested and subjected to ELISA to examine viral accumulation. Seven out of 21 accessions were confirmed to be 100 percent symptomless with no CMV accumulation (**Figures 3A,B**). In short, from the bioassay on *Capsicum* germplasm, we identified seven additional CMV-P1 resistance accessions that might be new sources of CMV resistance.

### Inheritance Study and Allelism Test of the Resistant Accessions

The seven resistant accessions identified above were crossed with the susceptible “Jeju” and “Lam32” to study the inheritance of CMV resistance and allelic relationship with *cmr2* (Kang et al., 2005). About 10 to 20 F_1_ plants generated from “seven resistant accessions × Jeju” were inoculated with CMV-P1 and scored for the disease symptoms at 25 dpi. All F_1_ hybrids challenged with the viral inoculum were susceptible, suggesting that the resistance to CMV-P1 is recessive in seven resistant accessions (**Supplementary Table S2**). To reveal the allelic relationship between the seven resistance accessions and *cmr2* gene, we performed the test cross between the seven resistant accessions and “Lam32” (*cmr2*/*cmr2*). F_1_ plants from “seven resistant accessions × Lam32” were challenged with CMV-P1 that revealed that all F_1_ plants were resistant to CMV-P1 (**Table 3**). Genetic complementation results showed that the CMV-P1 resistance in seven resistant accessions could be derived from the *cmr2* locus.

**Table 3:** CMV resistance in pepper accessions and in the progeny of crosses between these accessions and “Lam32”.

Parent lines and populations	Number of plants			Expected ratio (R:S)
	Total	Resistant (R)	Susceptible (S)	
Lam32	10	10	0	1:0
IT221660	7	7	0	1:0
IT221661	6	6	0	1:0
IT221885	10	10	0	1:0
IT236359	7	7	0	1:0
IT236402	6	6	0	1:0
IT248570	10	10	0	1:0
IT264081	10	10	0	1:0
F_1_ “ IT221660 × Lam32”	10	10	0	1:0
F_1_ “IT221661 × Lam32”	10	10	0	1:0
F_1_ “IT221885 × Lam32”	10	10	0	1:0
F_1_ “IT236359 × Lam32”	5	5	0	1:0
F_1_ “IT236402 × Lam32”	19	19	0	1:0
F_1_ “IT248570 × Lam32”	20	20	0	1:0
F_1_ “IT264081 × Lam32”	18	18	0	1:0

As the seven accessions were suspected to have the same resistance gene, we used “Affy4” to confirm the alleles for *cmr2*. This marker was used to analyze several of the F_1_ plants derived from the crosses of the resistant plants with “Jeju” along with the parents. All resistant accessions showed the same genotype as “Lam32” (**Supplementary Figure S3**), which indicated that the CMV resistant accessions harbor the same single recessive CMV resistance gene, *cmr2*.

## Discussion

The discovery of single resistance genes is important for the successful breeding of resistant cultivars as it is more manageable to integrate a single gene into susceptible elite lines than it is for multiple genes. To date, most of the identified CMV resistance genes in *Capsicum* spp. are controlled by QTLs, and *Cmr1* is the only single dominant gene that confers resistance to CMV (Kang et al., 2010). *Cmr1* restricts the movement of the virus from epidermal cells into deeper layers of host cells (Kang et al., 2010). However, a new isolate, CMV-P1, has a variation in the helicase domain of RNA1 and can overcome the resistance conferred by *Cmr1*. In fact, a recent study demonstrated that *Cmr1*-based resistance can be broken by several amino acid modifications in the virus (Choi et al., 2016). Therefore, our identification of *cmr2* provides significant value as a single resistance gene that can be implemented in pepper breeding programs.

We also developed SNP markers linked to the *cmr2* locus. The closest marker, Affy4, was located on chromosome 8 about 2.3 cM from *cmr2*. The developed marker is located on one side of the *cmr2* locus in “Lam32.” Thus, markers on the other side and more closely linked markers were needed to pinpoint the physical location of *cmr2*. However, developing more closely linked markers was challenging. First, *cmr2* is located in a highly repetitive sequence region. Sequence analysis of the contigs (Ctg78917, Ctg75352, and Ctg81356) that have an Affy4 sequence motif predicted a “ribosomal RNA intron-encoded homing endonuclease” in the region. Highly repetitive short sequences are a challenge for alignment and assembly that can cause errors when interpreting the results (Treangen and Salzberg, 2011). We tried to move incrementally through the contigs from Ctg81356 to develop markers closer than Affy4; however, 9 kb was the physical limit because of ambiguity in the sequences and the absence of contigs that share sequence similarities (**Figure 2**).

Marker development was further complicated by the chromosomal rearrangement between chromosomes 1 and 8 induced by reciprocal translocation. The cultivated *C. annuum* has a different genomic structure from that of the wild species of *C. annuum*, *C. chinense*, and *C. frutescens*. The translocated breakpoint between chromosomes 1 and 8 resulted from two ribosomal DNA clusters (R45S-B and R45S-C) (Tanksley et al., 1988; Wu et al., 2009; Park et al., 2014). From the chromosomal localization analysis of *cmr2* using the cmvAFLP marker, we found that cmvAFLP is linked to chromosome 1 on the “AC99” map; however, when the sequence information was used to query “CM334” genomic data, the marker was located on chromosome 8 (**Figure 2**). Although the *cmr2*-associated markers cmvAFLP and IBP160 are aligned on chromosome 8 of the “CM334” physical map, the closer marker Affy4 could not be aligned on any of the chromosomes (**Figure 2**). Studies using fluorescence *in situ* hybridization will be helpful to confirm the exact chromosomal location of *cmr2*.

In plants, recessive resistance results from the absence or impaired functions of essential host factors that interfere with the successful cycle of viral infection. Over the decades, a number of recessive resistance genes have been identified and characterized (Truniger and Aranda, 2009; Hashimoto et al., 2016). Most of the recessive resistance genes isolated to date are related to the eIF4E and eIF4G complex. *Arabidopsis thaliana* plants harboring the mutation in eIF (iso)4E displayed resistance to several Potyviruses including *Tobacco etch virus*, *Turnip mosaic virus*, *Lettuce mosaic virus*, and *Plum mosaic virus* (Maule et al., 2007). The genes *pvr1* in pepper, *mo1* in lettuce, and *sbm1* in pea were all identified to have mutations in *eIF4E* (Ruffel et al., 2002; Nicaise et al., 2003; Gao et al., 2004). Other mutations also known to affect multiplication and movement of viruses for viral infection include those in genes encoding seven-pass membrane proteins [Tobamovirus multiplication 1 (TOM1) and (TOM2); Yamanaka et al., 2002; Ishibashi and Ishikawa, 2016], a small GTP-binding ARF-family protein (ARL8; Nishikiori et al., 2011), a chloroplast phosphoglycerate kinase (cPGK2; Ouibrahim et al., 2014), cell-to-cell transporter [potyvirus VPg-interacting protein 1 and 2 (PVIP1) and (PVIP2); Dunoyer et al., 2004], and calcium sensors (Lewis and Lazarowitz, 2010).

Recessive resistance against CMV has also been identified in Arabidopsis. *CUM1* and *CUM2* that encode eIF4E and 4G, respectively, inhibit the cell-to-cell movement of CMV (Yoshii et al., 1998, 2004). Moreover, mutations in CmVPS41, the product of *cmv1*, confer resistance to CMV in *Cucumis melo.* The *CmVPS41* gene encodes a protein involved in membrane trafficking to the vacuole (Giner et al., 2017). The other CMV resistance gene, *cmv1*, have been also reported in melon. *cmv1* gene restricts systemic infection of specific strain of CMV, CMV-LS, preventing virus propagation from bundle sheath cells to phloem cells (Guiu-Aragonés et al., 2016). In this study, the CMV_FNY_-GFP signals were observed in mesophyll cells in the resistance plants “Lam32” whereas the GFP signals were detected in both epidermal and mesophyll cells in the susceptible plants “Jeju” (**Supplementary Figure S1**). It would be interesting to know if the resistance governed by *cmr2* is directly associated with the inhibition of cell-to-cell or systemic movement via examination of the occurrence of virus in systemic leaves with different time points. When we examined the mapped regions for genes that encode proteins involved in viral movement, none of them were mapped to the *cmr2* locus. Further studies are required to reveal the identity and the exact mechanism of resistance conferred by *cmr2*.

The availability of recessive resistance genes together with information on plant-virus protein interactions will provide new ways to develop novel resistance alleles in plants using gene editing technologies such as transcriptional activator-like effector nucleases (Cheng et al., 2015) and clustered regularly interspaced short palindromic repeats associated with endonuclease 9 (CRISPR/Cas9) (Doudna and Charpentier, 2014). Genome editing via the CRISPR/Cas9 system has been demonstrated to create novel resistance alleles in *eIF4E* (Belhaj et al., 2013; Nekrasov et al., 2013; Xie et al., 2014; Ali et al., 2015; Bortesi and Fischer, 2015; Jacobs et al., 2015; Chandrasekaran et al., 2016). Once we find the identity of the *cmr2* gene, this gene can be another target for genome editing.

We identified seven different accessions of *C. annuum* with resistance to CMV-P1 among 1000s of pepper germplasms (**Figure 3**). Surprisingly, allelism and inheritance tests revealed that the genes that govern resistance in these accessions are located at the same position as *cmr2*, which indicated that *cmr2* is a unique resistance gene for CMV-P1. It is also possible additional alleles exist in the identified resistant accessions, as was shown with the *pvr1* locus of pepper (Kang et al., 2005). To test whether these accessions have different alleles, the screened accessions need to be examined with various strains of CMV.

In summary, we found that *cmr2* is a single recessive resistance gene that confers resistance to CMV-P1 and we provided closely related markers for genotyping this gene, which will be useful in pepper breeding programs. Future research on *cmr2* will focus on investigation of the mechanism for CMV resistance in pepper.

## Author Contributions

B-CK, SC, and W-HK conceived and designed this study. SC, J-HL, W-HK, HH, S-WP, and E-HS performed the experiments. SC, J-HL, W-HK, JK, HH, S-WP, and J-KK analyzed the data. B-CK, SC, W-HK, and JK wrote the manuscript.

## Conflict of Interest Statement

The authors declare that the research was conducted in the absence of any commercial or financial relationships that could be construed as a potential conflict of interest.

## Footnotes

1 http://www.bioconductor.org/

2 http://peppergenome.snu.ac.kr
