# Supplementary material for: Identification of Cucumber mosaic resistance 2 (cmr2) That Confers Resistance to a New Cucumber mosaic virus Isolate P1 (CMV-P1) in Pepper (Capsicum spp.)
Source: Front Plant Sci. 2018 Aug 21;9:1106. doi: 10.3389/fpls.2018.01106 (PMC6110927; doi:10.3389/fpls.2018.01106)
Supplement: Supplementary file 1 [file Data_Sheet_1.PDF]

Supplementary Material

Identification of *Cucumber mosaic resistance 2* (*cmr2*) that confers resistance to a new *Cucumber mosaic virus* isolate P1 (CMV-P1) in pepper (*Capsicum* spp)

Seula Choi, Joung-Ho Lee, Won-Hee Kang, Joonyup Kim, Hoang Ngoc Huy, Sung-Woo Park, Eun-Ho Son, Jin-Kyung Kwon, and Byoung-Cheorl Kang*

*** Correspondence:** Corresponding Author: bk54@snu.ac.kr

1. Supplemental files

Figure legends

Figure S1. Localization of CMV-GFP in *C. annuum* 'Jeju' and 'Lam32'. CMV_{FNY}-GFP was inoculated into the cotyledons of 'Jeju' and 'Lam32' were inoculated with CMV_{FNY}-GFP. GFP fluorescence was observed by confocal laser scanning microscopy in two tissues, the epidermal layer (A, C, E, G) and the mesophyll layer (B, D, F, H) at 2 dpi (A-D) and 6 dpi (E-H) (Kang et al., 2010). All images are "Z projections" of 7–15 slices. Green signal indicates the expression of GFP and red signal indicates autofluorescence. Scale bars = 50 μm. All experiments were repeated three separate times with three biological replicates. Representative images are shown.

Figure S2. Conversion of the BSA-AFLP marker into the SNP-based HRM marker. (A) Gel image from AFLP analysis. Yellow arrow represents the polymorphism between the 12 resistant and 13 susceptible pooled samples. The polymorphic AFLP fragment (arrow) was retrieved from the amplicon of the susceptible control 'Jeju'. (B) Sequence alignment among the 'Jeju' AFLP fragment and dideoxy sequencing result of 'Jeju' and 'Lam32'. Red underlines indicate the primer sites of the cmvAFLP HRM amplicon. Red boxes represent the SNP sites in the HRM amplicon region. (C) Normalized melting curve plot of the cmvAFLP HRM marker.

Figure S3. Kompetitive Allele-Specific PCR (KASP) genotyping analysis of the *cmr2* marker, Affy4. X-axis represents FAM fluorescence (465-510 nm) and Y-axis represents HEX fluorescence (533-580 nm) on an endpoint fluorescence scatter plot. Susceptible

samples including 'Jeju' control (yellow), heterozygous samples including 'JejuxLam32' F_1 control (red), and resistant samples including 'Lam32' control (blue).

Table legends

Table S1. Summary of *Capsicum* germplasm screening against the CMV-P1 isolate.

Table S2. Inheritance study of the new source of resistance against the CMV-P1.

2. Supplementary Figures and Tables

2.1 Supplementary Figures

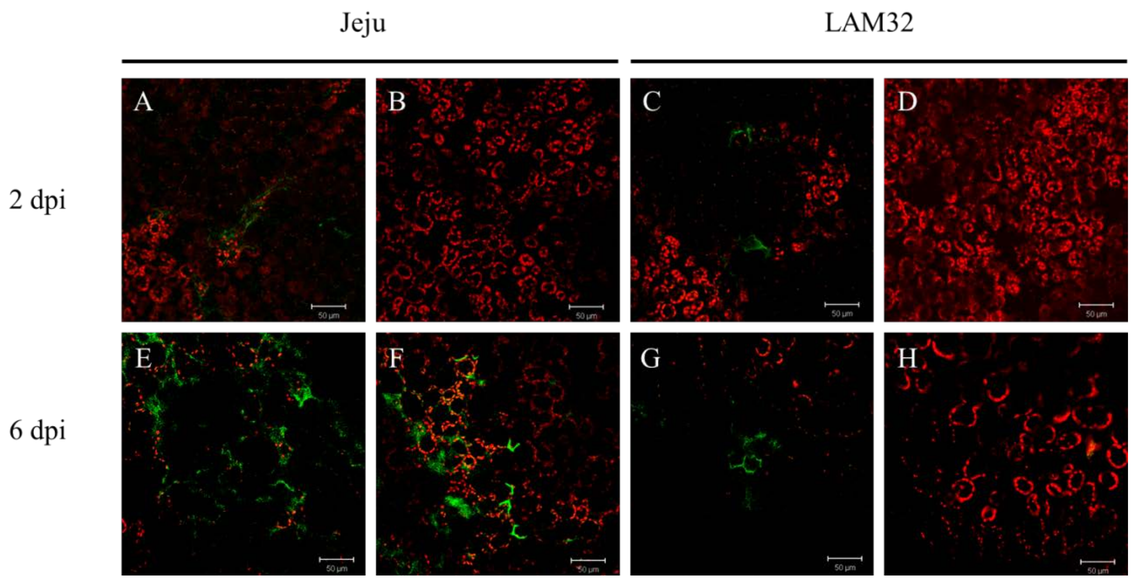

Figure S1. Localization of CMV-GFP in *C. annuum* 'Jeju' and 'Lam32'. CMV_{FNY}-GFP was inoculated into the cotyledons of 'Jeju' and 'Lam32' were inoculated with CMV_{FNY}-GFP. GFP fluorescence was observed by confocal laser scanning microscopy in two tissues, the epidermal layer (A, C, E, G) and the mesophyll layer (B, D, F, H) at 2 dpi (A-D) and 6 dpi (E-H) (Kang et al., 2010). All images are "Z projections" of 7–15 slices. Green signal indicates the expression of GFP and red signal indicates autofluorescence. Scale bars = 50 μm. All experiments were repeated three separate times with three biological replicates. Representative images are shown.

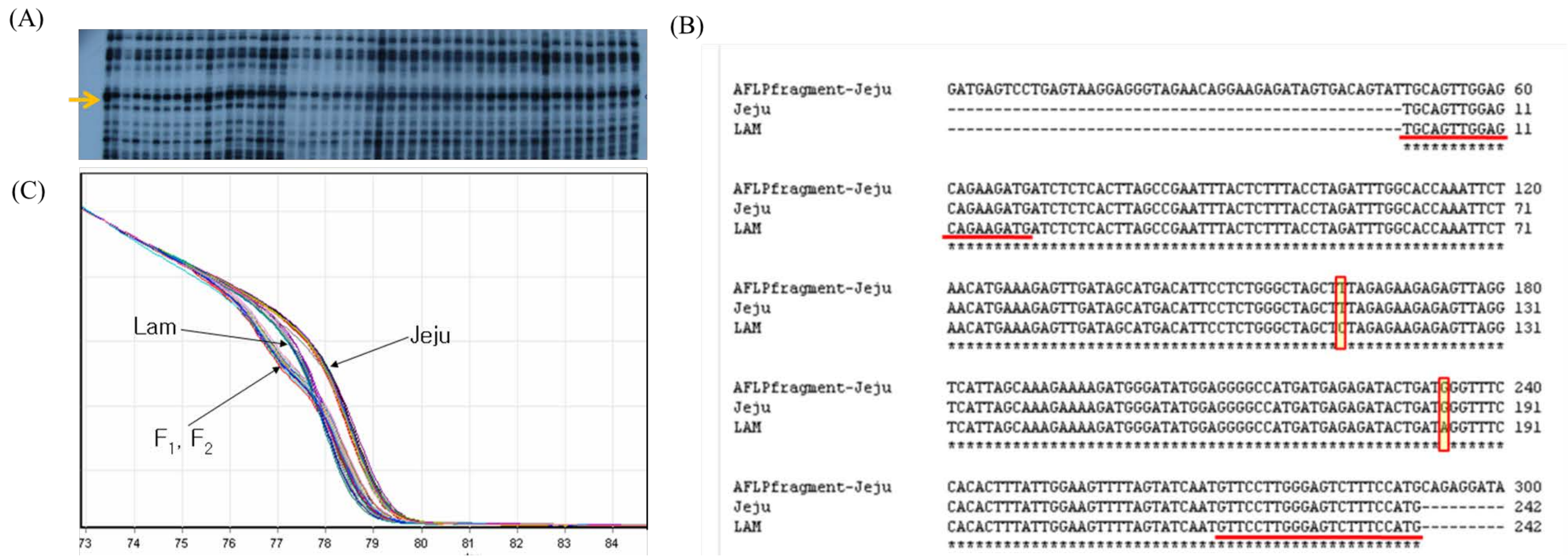

Figure S2. Conversion of the BSA-AFLP marker into the SNP-based HRM marker. (A) Gel image from AFLP analysis. Yellow arrow represents the polymorphism between the 12 resistant and 13 susceptible pooled samples. The polymorphic AFLP fragment (arrow) was retrieved from the amplicon of the susceptible control 'Jeju'. (B) Sequence alignment among the 'Jeju' AFLP fragment and dideoxy sequencing result of 'Jeju' and 'Lam32'. Red underlines indicate the primer sites of the cmvAFLP HRM amplicon. Red boxes represent the SNP sites in the HRM amplicon region. (C) Normalized melting curve plot of the cmvAFLP HRM marker.

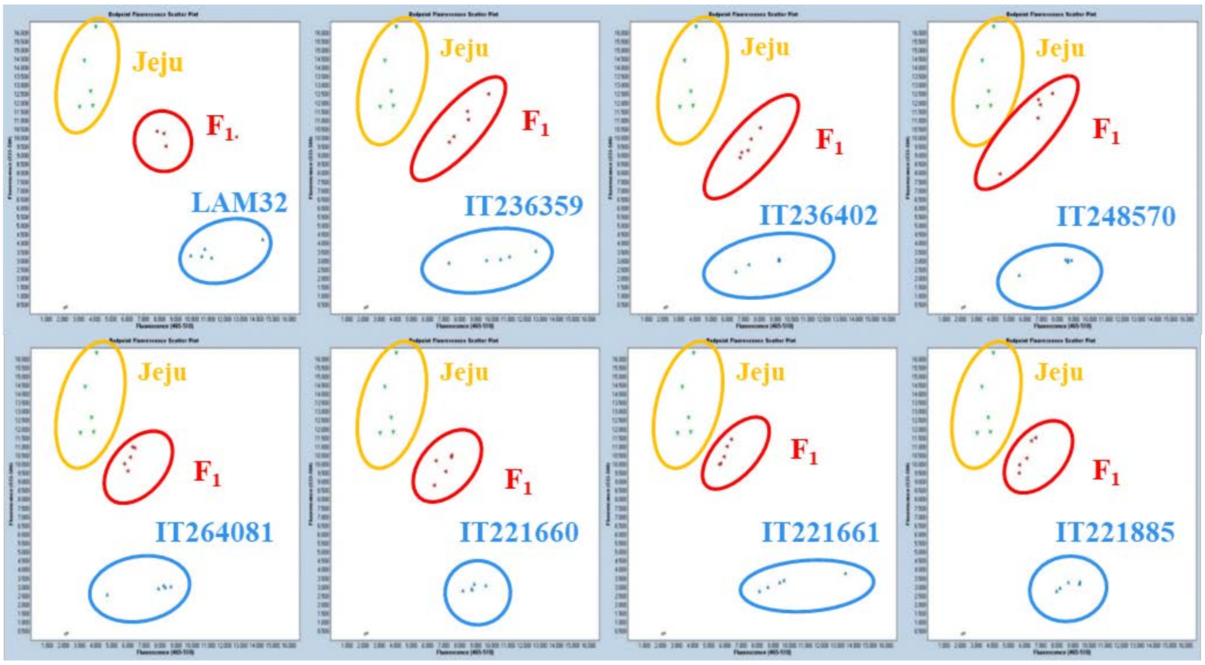

Figure S3. Kompetitive Allele-Specific PCR (KASP) genotyping analysis of the *cmr2* marker, Affy4. X-axis represents FAM fluorescence (465-510 nm) and Y-axis represents HEX fluorescence (533-580 nm) on an endpoint fluorescence scatter plot. Susceptible samples including 'Jeju' control (yellow), heterozygous samples including 'JejuxLam32' F_1 control (red), and resistant samples including 'Lam32' control (blue).

2.2 Supplementary Tables

Table S1. Summary of *Capsicum* germplasm screening against the CMV-P1 isolate.

Capsicum species	Total number of accessions	Number of accessions At 21 dpi	
		Susceptible	Resistant
Capsicum annuum	2594	2576	28
Capsicum baccatum	248	248	0
Capsicum chinense	208	208	0
Capsicum frutescens	146	143	3
Capsicum pubescens	2	2	0
Total	4197	4176	21

Table S2. Inheritance study of the new source of resistance against CMV-P1.

Parent lines and populations	Number of plants			Expected ratio (R : S)
	Total	Resistant (R)	Susceptible (S)	
Jeju	10	0	10	0:1
IT221660	7	7	0	1:0
IT221661	6	6	0	1:0
IT221885	10	10	0	1:0
IT236359	7	7	0	1:0
IT236402	6	6	0	1:0
IT248570	10	10	0	1:0
IT264081	10	10	0	1:0
F_1 ‘ IT221660×Jeju’	10	0	10	0:1
F_1 ‘IT221661×Jeju’	10	0	10	0:1
F_1 ‘IT221885×Jeju’	10	0	10	0:1
F_1 ‘IT236359×Jeju’	5	0	5	0:1
F_1 ‘IT236402×Jeju’	18	0	18	0:1
F_1 ‘IT248570×Jeju’	19	0	19	0:1
F_1 ‘IT264081×Jeju’	20	0	20	0:1
